# Epidemiology and clinical features of Hymenoptera stings in East Azerbaijan, northwestern Iran

**DOI:** 10.1371/journal.pone.0353652

**Published:** 2026-07-16

**Authors:** Hadi Rezaei, Amirhossein Babapour, Maryam Porkar Khatibi, Pouya Golshaniniya, Ali Pourhassan, Ehsan Mirzaaghazadeh, Farzad Rahmani, Abdollah Badzohreh, Simin Khayatzadeh, Madineh Abbasi

**Affiliations:** 1 Student Research Committee, Tabriz University of Medical Sciences, Tabriz, Iran; 2 Emergency and Trauma Care Research Center, Tabriz University of Medical Sciences, Tabriz, Iran; 3 Department of Disease Prevention and Control, Health Deputy, Maragheh University of Medical Sciences, Maragheh, Iran; 4 Province Health Center, Tabriz University of Medical Sciences, Tabriz, Iran; 5 Social Determinants of Health Research Center, Tabriz University of Medical Sciences, Tabriz, Iran; 6 Infectious and Tropical Diseases Research Center, Sina Hospital, Tabriz University of Medical Sciences, Tabriz, Iran; Tsinghua University, CHINA

## Abstract

**Background:**

Stings caused by Hymenoptera including honey bees, wasps, and hornets represent a growing global and national health concern with outcomes ranging from mild local reactions to severe systemic complications. Despite their significance, region-specific epidemiological data from Iran remain limited. This study aimed to investigate the demographic characteristics, anatomical distribution, seasonal patterns, clinical features, and hospitalization-related factors among Hymenoptera sting victims in northwestern Iran.

**Methods:**

A retrospective descriptive-analytical study was conducted between 2021 and 2024 in East Azerbaijan Province, Iran, using medical records from hospitals affiliated with Tabriz University of Medical Sciences. A total of 225 eligible patients with confirmed or clinically suspected Hymenoptera stings were analyzed through a census-based approach. Demographic (including age, sex, and occupational status), epidemiological, and clinical variables were collected via structured forms. Descriptive statistics were applied. Chi-square or exact tests were used to assess associations between categorical variables, and multivariable logistic regression was performed to identify independent predictors of hospitalization. Statistical significance was set at p < 0.05.

**Results:**

Among 225 cases, 71.6% were male, and the mean age was 30.99 ± 18.49 years, showing a bimodal distribution peaking in children aged <10 years and adults aged 30–49 years. A highly significant association was found between gender and occupation (p < 0.001); females were predominantly housewives, males were distributed mainly among self-employed individuals, students, farmers, and manual workers. Urban areas accounted for 53.3% of incidents, and summer was the peak season (52.4%). The head and neck were the most affected sites (44.9%), with a significant gender disparity: males were more frequently stung on the head/neck, whereas females were more frequently stung on the limbs (p < 0.001). Hospitalization was required in 28.0% of patients. In multivariable logistic regression, pain (OR = 40.43, 95% CI: 12.56–130.15), severe muscle pain (OR = 77.56, 95% CI: 12.97–463.89), and local swelling/redness (OR = 16.05, 95% CI: 3.96–65.06) were independently associated with hospitalization. Male gender showed a non-significant trend toward higher odds of hospitalization (OR = 3.06, p = 0.067).

**Conclusion:**

Hymenoptera stings in northwestern Iran are shaped by a complex interplay of ecological, occupational, and socio-cultural factors. The distinct anatomical sting patterns may be influenced by regional dress codes and gender-specific activities, suggest that preventive strategies should address both domestic and occupational exposure settings. Public health interventions should focus on habitat management in residential areas, awareness among households, and protective clothing for outdoor workers to reduce the burden of envenomation.

## Introduction

Stings caused by Hymenoptera – encompassing bees, wasps, and hornets – represent a significant global public health concern. In some countries such as Brazil, these incidents rank as the second leading cause of human envenomation after drug poisoning. Epidemiological data indicate a rising global trend in Hymenoptera sting cases; for instance, between 2013 and 2023, more than 206,000 cases were recorded in Brazil alone [1]. Although fatal outcomes are relatively uncommon, a comprehensive epidemiological study across Europe reported 1,691 sting-related deaths between 1994 and 2016, predominantly among middle-aged men [2].

In Iran, several studies have reported Hymenoptera stings with varying clinical outcomes. For example, Bemanian et al. documented 201 cases of sting-induced anaphylaxis in Gorgan [3]. Sedighi et al. reported a severe toxic reaction following multiple hornet stings in Kerman Province [4]. Dehghani et al. provided a comprehensive overview of venomous arthropods in Iran and neighboring regions [5]. These findings highlight the diversity of Hymenoptera species and the importance of region-specific epidemiological data.

The clinical spectrum of venom-induced reactions is broad, ranging from mild local manifestations such as pain, erythema, and swelling to severe systemic complications. Anaphylaxis constitutes the most severe outcome and is considered one of the most life-threatening allergic emergencies in adults. This reaction is typically mediated by an IgE-driven Type I hypersensitivity mechanism, whereby the immune system mounts an exaggerated response [6]. Furthermore, in cases of multiple stings, systemic toxic reactions [6] may occur due to the high volume of venom injected, potentially leading to acute renal failure, rhabdomyolysis, hepatic injury, and multi-organ dysfunction [7–9]. Delayed and unusual complications have also been documented, including hepatitis, Kounis syndrome (allergic myocardial infarction), and neurological or coagulation disorders [9,10]. Hymenoptera venom sensitization may be detectable in nearly 26% of adults, underscoring the critical role of a detailed clinical history in the initial risk assessment [11].

Global epidemiological studies have identified recurring patterns. A consistent male predominance among victims has been reported in studies from Brazil, Europe, and Iran, commonly attributed to greater male participation in outdoor occupational and recreational activities [1,2,8,11,12]. A pronounced seasonal trend has also been established, with peak incidence during warmer months such as summer and autumn [8,9]. Most stings occur in urban areas, with the head and neck representing the most frequently affected anatomical sites [8,12]. Additionally, environmental factors such as climate change, deforestation, and urbanization are altering insect behavior and increasing the frequency of human–insect encounters [1,10].

Despite the significance of the issue, epidemiological data detailing demographic characteristics, geographic distribution among sting victims remain limited—particularly in rural and semi-industrial regions of Iran. This gap is not unique to Iran; European authors have similarly highlighted the need for region-specific epidemiological data [2]. For example, a cross-sectional study in rural areas of Gorgan documented 201 cases of sting-induced anaphylaxis, emphasizing both the frequent systemic involvement and the critical role of rural healthcare infrastructure [11]. However, that study focused exclusively on the most severe reaction (anaphylaxis) and did not encompass the full clinical spectrum of sting-related complications. To date, no structured investigation has analyzed the clinical and demographic patterns of Hymenoptera sting victims in northwestern Iran. This study aims to address this knowledge gap by examining the demographic characteristics, anatomical sites of stings, seasonal distribution, clinical manifestations, and hospitalization-related factors among affected patients.

## Materials and methods

### Study design

**Fig 1:**
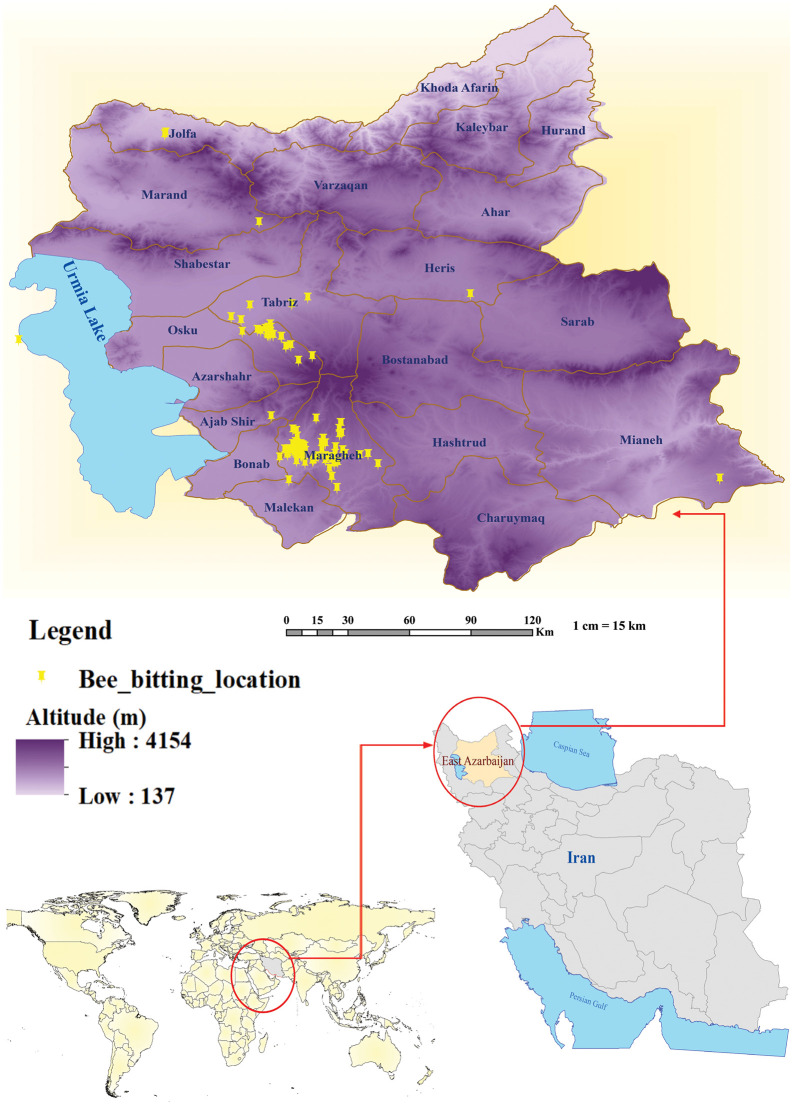
The map of study areas in East Azerbaijan, northwestern Iran. Map created by the authors using ArcGIS Desktop (version 10.5; ESRI, Redlands, CA, USA). The base map was sourced from OpenStreetMap (https://www.openstreetmap.org), which is available under the Open Database License (ODbL). Map data is copyrighted by OpenStreetMap contributors.

This descriptive-analytical study was conducted from January 2021 to December 2024, in East Azerbaijan Province, northwestern Iran. Data were collected from medical records of patients with Hymenoptera sting incidents who presented to emergency departments and hospitals affiliated with Tabriz University of Medical Sciences in Maragheh, Osku, and Ajabshir Counties (**Fig 1**). The anonymized medical record data were accessed for research purposes between February 15 and April 20, 2025.

### Study population

The study included all patients who presented with documented or clinically suspected Hymenoptera stings during the study period. A total of 225 cases met the inclusion criteria and were analyzed. Inclusion criteria included a documented or clinically suspected diagnosis of Hymenoptera sting recorded in the patient’s medical file and availability of core demographic and clinical information.

### Data collection and variables

Variables were grouped into demographic characteristics (age, gender, and place of residence), epidemiological characteristics (season, geographic setting of the sting, and time interval between sting and hospital visit), and clinical features including anatomical site of the sting, type and severity of symptoms, underlying comorbidities, and treatments. The clinical symptoms were local (pain, swelling, erythema, numbness) and systemic (urticaria, generalized itching, shortness of breath, nausea, and altered consciousness). The duration of hospital stays and hospitalization status (inpatient vs. outpatient) were recorded. Personal identifiers were removed prior to data extraction to maintain confidentiality. Due to the retrospective nature of the study and reliance on hospital registry data, exact species-level identification was not available. Classification was limited to clinically suspected Hymenoptera stings based on medical records and patient reports.

### Data analysis

Data were analyzed using SPSS version 26. Descriptive statistics were used for all variables. Continuous variables, including age and time delay, were reported as mean ± standard deviation (SD). Categorical variables, including age group, gender, anatomical site, season, occupation, and location, were expressed as frequencies and percentages. Chi-square tests were conducted to assess associations between gender and key variables, as well as between hospitalization status and demographic, epidemiological, and clinical variables. To identify independent predictors of hospitalization, multivariable logistic regression analysis was performed with hospitalization status as the dependent variable. Age was entered as a continuous variable, and gender, anatomical site, pain, severe muscle pain, and local swelling/redness were entered as independent variables. Adjusted odds ratios (ORs) with 95% confidence intervals (CIs) were calculated. Variables with very low frequencies or complete/separation issues, including itching, numbness/muscle cramps, acute systemic symptoms, weakness/hypotension, and geographic location, were not included in the final multivariable model. Model fit was assessed using the Hosmer–Lemeshow goodness-of-fit test. A p-value < 0.05 was considered statistically significant.

### Ethical considerations

This study was reviewed and approved by the Ethics Committee of Tabriz University of Medical Sciences (Ethics Code: IR.TBZMED.AEC.1402.080). The study was conducted in accordance with the ethical principles of the Declaration of Helsinki. Given the retrospective nature of the study and the use of anonymized medical records, the requirement for informed consent to participate was waived by the Ethics Committee of Tabriz University of Medical Sciences, in accordance with national research ethics regulations. All patient data were fully anonymized prior to analysis, and no identifiable personal information was accessed or included in the study.

## Results

### Demographic characteristics

In this study, data from 225 patients presenting with a diagnosis of a Hymenoptera sting (bee, wasp, or hornet) were analyzed. Of these, 161 (71.6%) were male and 64 (28.4%) were female, indicating a male-to-female ratio of approximately 2.5 to 1.

**Fig 2:**
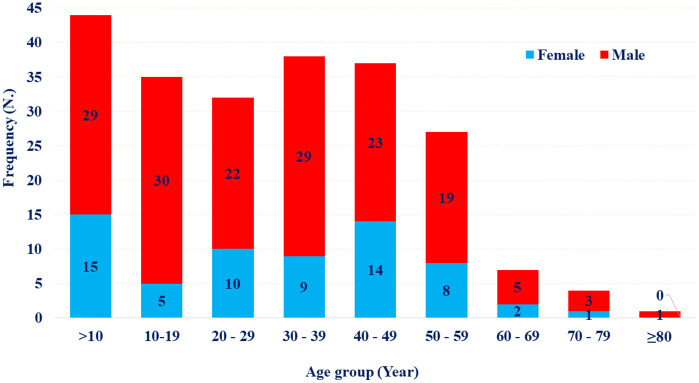
Hymenoptera sting incidents by gender and age group in East Azerbaijan Province, Iran (2021-2024).

The mean age of the patients was 30.99 ± 18.49 years, with an age range spanning from 1 to 82 years. An analysis of the age frequency distribution revealed a bimodal pattern. The first and largest peak of incidence was observed in the 0–9-year age group, accounting for 19.6% of cases. A second peak was noted in the 30–39 and 40–49-year age groups (16.9% and 16.4%, respectively) (**Fig 2**). The chi-square analysis revealed no statistically significant differences between age groups and gender in the context of Hymenoptera stings (χ² = 6.481, df = 7, p = 0.485).

Regarding occupational status, a highly significant association was observed between gender and occupation (χ² = 144.281, df = 6, p < 0.001). As shown in Table 1, there was a distinct gender-based distribution: female patients were predominantly housewives (65.6%), whereas male patients were distributed across various occupations, with the highest frequencies among self-employed individuals (30.4%), students (24.8%), farmers (15.5%), and manual workers (13.7%).

**Table 1:** Distribution of occupational categories, geographic location, and anatomical site by gender among patients with Hymenoptera stings in East Azerbaijan Province, Iran (2021-2024).

Variables		Gender				χ²	df	P-value	Total	
		Female		Male						
		n	%	n	%				n	%
Occupation	**Self-Employed**	**0**	**0**	**49**	**30.4**	144.281	**6**	< 0.001	**49**	**21.8**
	**Housewife**	**42**	**65.6**	**0**	**0**				**42**	**18.7**
	**Farmer**	**0**	**0**	**25**	**15.5**				**25**	**11.1**
	**Student**	**14**	**21.9**	**40**	**24.8**				**54**	**24.0**
	**Manual workers**	**0**	**0**	**22**	**13.7**				**22**	**9.8**
	**Employee**	**4**	**6.3**	**13**	**8.1**				**17**	**7.6**
	**Child**	**4**	**6.3**	**12**	**7.5**				**16**	**7.1**
Geographic location	**Desert**	**0**	**0**	**7**	**4.3**	4.167	2	0.120	**7**	**3.1**
	**Rural**	**25**	**39.1**	**73**	**45.3**				**98**	**43.6**
	**Urban**	**39**	**60.9**	**81**	**50.3**				**120**	**53.3**
Anatomical site	**Head and neck**	18	28.1	83	51.6	**32.609**	**3**	< 0.001	101	44.9
	**Hand**	23	35.9	52	32.3				75	33.3
	**Legs**	19	29.7	7	4.3				26	11.6
	**Torso**	4	6.3	19	11.8				23	10.2
Total		**64**	**100**	**161**	**100**				**225**	**100**

### Spatiotemporal distribution

The majority of sting incidents occurred in urban areas (53.3%, n = 120), followed by rural regions (43.6%, n = 98) and desert areas (3.1%, n = 7). Based on the Monte Carlo exact test (conducted due to sparse cell counts in desert categories), there was no statistically significant association between gender and the geographic location of the sting (χ² = 4.167, df = 2, p = 0.120). Although all 7 cases in desert areas involved male patients, the overall geographic distribution did not differ significantly between genders (Table 1).

**Fig 3:**
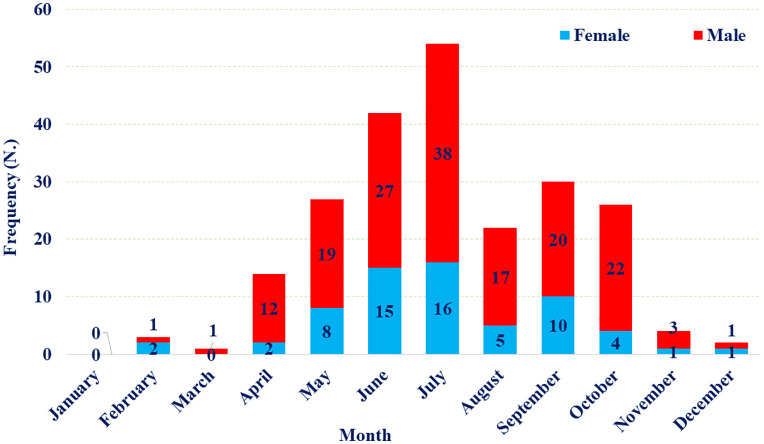
Monthly Trend of Hymenoptera Sting Incidents by Gender in East Azerbaijan Province, Iran (2021-2024).

The monthly trend of Hymenoptera sting incidents by gender is illustrated in **Fig 3**. Overall, males experienced a higher frequency of stings than females, with the most notable differences observed during the summer months, particularly June, July, and August.

Seasonal analysis showed that Hymenoptera stings were strongly influenced by the season. More than half of the cases (52.4%) occurred during the summer. Following summer were fall (26.7%), spring (18.7%), and winter (2.2%). No statistically significant association was found between gender and the season of sting occurrence (p = 0.323, χ² = 3.486). The seasonal pattern was similar for both genders, with the incidence peaking in the summer for both males (50.9%) and females (56.3%).

### Anatomical site of sting

The most commonly affected area was the head and neck, which accounted for 44.9% of all cases. This was followed by the hands and legs. The anatomical distribution of stings varied significantly between genders (χ^2^ = 32.61, df = 3, p < 0.001). For male patients, the head and neck was the most frequently affected region (51.6%), whereas females experienced a higher proportion of stings on their legs (29.7% vs. 4.3% in males) and hands (35.9% vs. 32.3% in males). Detailed frequencies and statistical comparisons are summarized in Table 1.

### Factors associated with hospitalization

The demographic, epidemiological, and clinical characteristics of hospitalized and outpatient patients are compared in Table 2. A total of 63 patients (28.0%) were recorded as hospitalized, although most represented short-term admissions and were discharged within 6 hours; the remaining 162 patients (72.0%) were treated as outpatients. Among demographic and epidemiological variables, anatomical site (p = 0.016) and geographic location (p < 0.001) were significantly associated with hospitalization in univariate analysis. Notably, all 7 cases recorded in desert areas required hospitalization, compared to 29.6% in rural and 23.3% in urban areas. Other demographic factors, including gender (p = 0.105), age group (p = 0.129), and occupation (p = 0.338), did not significantly influence the rate of hospitalization.

**Table 2:** Comparison of demographic, epidemiological, and clinical characteristics between hospitalized and outpatient patients with Hymenoptera stings (n = 225).

Variable	Category	Hospitalized n = 63, n (%)	Outpatient n = 162, n (%)	Total n = 225, n (%)	P-value
**Gender**	**Male**	50 (79.4)	111 (68.5)	161 (71.6)	0.105
	**Female**	13 (20.6)	51 (31.5)	64 (28.4)	
**Age group** **(years)**	**<10**	9 (14.3)	35 (21.6)	44 (19.6)	0.129
	**10–19**	12 (19.0)	23 (14.2)	35 (15.6)	
	**20–29**	12 (19.0)	20 (12.3)	32 (14.2)	
	**30–39**	13 (20.6)	25 (15.4)	38 (16.9)	
	**40–49**	7 (11.1)	30 (18.5)	37 (16.4)	
	**50–59**	4 (6.3)	23 (14.2)	27 (12.0)	
	**60–69**	3 (4.8)	4 (2.5)	7 (3.1)	
	**70 and above**	3 (4.8)	2 (1.2)	5 (2.2)	
**Occupation**	**Child**	4 (6.3)	12 (7.4)	16 (7.1)	0.338
	**Employee**	3 (4.8)	14 (8.6)	17 (7.6)	
	**Farmer**	5 (7.9)	20 (12.3)	25 (11.1)	
	**Housewife**	9 (14.3)	33 (20.4)	42 (18.7)	
	**Self‑employed**	20 (31.7)	29 (17.9)	49 (21.8)	
	**Student**	15 (23.8)	39 (24.1)	54 (24.0)	
	**Worker**	7 (11.1)	15 (9.3)	22 (9.8)	
**Anatomical site**	**Head and neck**	23 (36.5)	78 (48.1)	101 (44.9)	0.016
	**Hand**	31 (49.2)	44 (27.2)	75 (33.3)	
	**Legs**	5 (7.9)	21 (13.0)	26 (11.6)	
	**Torso**	4 (6.3)	19 (11.7)	23 (10.2)	
**Geographic location**	**Desert**	7 (11.1)	0 (0.0)	7 (3.1)	<0.001
	**Rural**	29 (46.0)	69 (42.6)	98 (43.6)	
	**Urban**	27 (42.9)	93 (57.4)	120 (53.3)	
**Pain**	**Yes**	30 (47.6)	6 (3.7)	36 (16.0)	<0.001
	**No**	33 (52.4)	156 (96.3)	189 (84.0)	
**Severe muscle pain**	**Yes**	12 (19.0)	2 (1.2)	14 (6.2)	<0.001
	**No**	51 (81.0)	160 (98.8)	211 (93.8)	
**Local swelling/redness**	**Yes**	17 (27.0)	4 (2.5)	21 (9.3)	<0.001
	**No**	46 (73.0)	158 (97.5)	204 (90.7)	
**Itching**	**Yes**	9 (14.3)	1 (0.6)	10 (4.4)	<0.001
	**No**	54 (85.7)	161 (99.4)	215 (95.6)	
**Numbness/muscle cramp**	**Yes**	6 (9.5)	0 (0.0)	6 (2.7)	<0.001
	**No**	57 (90.5)	162 (100.0)	219 (97.3)	
**Acute systemic symptoms**	**Yes**	6 (9.5)	0 (0.0)	6 (2.7)	<0.001
	**No**	57 (90.5)	162 (100.0)	219 (97.3)	
**Weakness/hypotension**	**Yes**	1 (1.6)	0 (0.0)	1 (0.4)	0.280
	**No**	62 (98.4)	162 (100.0)	224 (99.6)	
**Skin rash**	**Yes**	1 (1.6)	0 (0.0)	1 (0.4)	0.280
	**No**	62 (98.4)	162 (100.0)	224 (99.6)	
**Comorbidity**	**Yes**	2 (3.2)	3 (1.9)	5 (2.2)	0.621
	**No**	61 (96.8)	159 (98.1)	220 (97.8)	

* Data are presented as n (%). Percentages were calculated within hospitalization status groups. P-values were obtained using the chi-square test or Fisher’s exact/Fisher–Freeman–Halton exact test when appropriate. For variables with sparse cell counts, exact or Monte Carlo estimates were used.

Clinical manifestations showed strong associations with hospital admission in univariate analysis. Pain was significantly more frequent among hospitalized patients than outpatients (47.6% vs. 3.7%, p < 0.001). Similarly, severe muscle pain (19.0% vs. 1.2%), local swelling/redness (27.0% vs. 2.5%), and itching (14.3% vs. 0.6%) were significantly more frequent in the hospitalized group (all p < 0.001). Furthermore, all patients who experienced numbness/muscle cramps or acute systemic symptoms were among those hospitalized (p < 0.001). Only 2.2% of cases had a documented history of underlying comorbidities, including chronic pulmonary disease, diabetes mellitus, and cardiovascular disease. The presence of comorbidities did not show a statistically significant relationship with the need for hospitalization (p = 0.621).

Although geographic location and itching were significantly associated with hospitalization in univariate analysis, they were not included in the final multivariable logistic regression model. Geographic location was excluded because all desert-area cases were hospitalized, resulting in complete separation, while itching was excluded due to its low frequency and sparse cell distribution. A multivariable logistic regression analysis was performed to identify factors independently associated with hospitalization after Hymenoptera stings. The regression model was statistically significant (Omnibus χ²(8) = 124.175, p < 0.001), with a Nagelkerke R^2^ of 0.611. The Hosmer–Lemeshow test was non-significant (χ²(8) = 7.677, p = 0.466), suggesting adequate model calibration. The model correctly classified 89.3% of cases.

In the multivariable model, pain, severe muscle pain, and local swelling/redness were independent predictors of hospitalization. Patients presenting with pain was strongly associated with increased odds of hospitalization compared with those without pain (OR = 40.43, 95% CI: 12.56–130.15, p < 0.001). Severe muscle pain was associated with 77.6 times higher odds of hospitalization (OR = 77.56, 95% CI: 12.97–463.89, p < 0.001), while local swelling/redness increased the odds of hospitalization by 16.1 times (OR = 16.05, 95% CI: 3.96–65.06, p < 0.001). Male gender showed a non-significant trend toward higher odds of hospitalization compared with female gender (OR = 3.06, 95% CI: 0.92–10.12, p = 0.067). Age and anatomical site were not independently associated with hospitalization (Table 3).

**Table 3:** Multivariable logistic regression analysis of factors associated with hospitalization among patients with Hymenoptera stings (N = 225).

Variable	B	S.E.	Wald	df	p-value	Odds Ratio (OR)	95% Confidence Interval (CI)
**Age** (years)	−0.007	0.012	0.360	1	0.549	0.993	0.969–1.017
**Gender** (Male vs. Female*)	1.118	0.611	3.351	1	0.067	3.058	0.924–10.121
**Anatomical Site** (Overall)			5.450	3	0.142		
Torso vs. Head & Neck*	−1.164	1.002	1.349	1	0.245	0.312	0.044–2.226
Hand vs. Head & Neck*	0.758	0.489	2.396	1	0.122	2.133	0.817–5.567
Legs vs. Head & Neck*	−0.199	0.882	0.051	1	0.821	0.819	0.146–4.611
**Pain** (Yes vs. No*)	3.700	0.597	38.465	1	**< 0.001**	40.429	12.559–130.149
**Severe Muscle Pain** (Yes vs. No*)	4.351	0.913	22.731	1	**< 0.001**	77.556	12.966–463.893
**Local Swelling/Redness** (Yes vs. No*)	2.776	0.714	15.111	1	**< 0.001**	16.051	3.960–65.057
**Constant**	−3.167	0.740	18.335	1	< 0.001	0.042	

* Reference categories were female for gender, head and neck for anatomical site, and no for pain, severe muscle pain, and local swelling/redness.

**Model Fit Indices:**

• Omnibus test of model coefficients: χ²(8) = 124.175, p < 0.001.

• Nagelkerke R^2^ = 0.611.

• Hosmer–Lemeshow test: χ²(8) = 7.677, p = 0.466.

• Overall classification accuracy = 89.3%.

### Delay before medical presentation

Data on the time interval between the sting and hospital presentation were available for 63 patients. Among these patients, 46.0% sought medical care more than 6 hours after the sting, whereas 33.3% presented within the first hour of the incident.

### Therapeutic interventions

All patients received at least one form of treatment. Corticosteroids were the most frequently administered therapy (75.0%), followed by antihistamines (31.6%) and wound cleansing (13.3%).

## Discussion

Hymenoptera stings represent a frequent cause of medical attention worldwide, producing a spectrum of outcomes from localized inflammatory reactions to severe anaphylaxis and, in rare cases, fatal multi-organ dysfunction. Epidemiological profiles differ substantially between regions, reflecting ecological conditions, seasonal patterns, species distribution, and cultural or occupational exposures [12–14].

Globally, most reports point to young adults as the highest-risk group, reflecting their economic activity and outdoor exposure [8,12,14]. Against this backdrop, our study revealed a distinctive bimodal pattern, with peaks in childhood and middle age. This differs from findings in India [9], where a predominance in older adults (>45 years) was observed, and from several Brazilian studies that reported an earlier peak in the 20–29 age group [8,12]. These discrepancies likely stem from regional differences in workforce structure and population demographics. Overall, our findings support the consistently reported elevated risk among economically active adults [8,12,15].

Age is a critical determinant of clinical presentation and outcomes. While our study highlighted a significant peak in children under 10 years and adults aged 30–50, international research identifies unique risks at both ends of the age spectrum. For instance, studies in Brazil have shown that individuals over 60 years face a significantly higher risk of severe systemic outcomes and mortality due to pre-existing comorbidities and diminished physiological reserve [15–17]. Conversely, the clinical significance of pediatric cases is characterized by a high frequency of incidents but often lower severity. While some studies in Thailand suggested that age under 15 might have a protective value against severe anaphylaxis [7,17], other research emphasizes that children remain highly vulnerable due to their smaller body mass and inquisitive behaviors, which can lead to multiple stings [7,17,18]. In our cohort, the childhood peak (19.6%) likely reflects behavioral exposure during outdoor play. Recognizing these age-specific patterns is essential for clinical triage, as pediatric patients require vigilant monitoring for systemic reactions, even if the immediate risk of severe anaphylaxis is statistically lower than in older adults.

Occupational distributions differed markedly between male and female patients, suggesting potentially different exposure settings. While male cases were distributed among outdoor-oriented occupations such as self-employment (30.4%), farming (15.5%), and manual labor (13.7%), female patients were predominantly housewives (65.6%). This pattern is consistent with Yu et al. (2025), who reported that males are more frequently stung during work-related outdoor activities [19]. However, the high prevalence of stings among housewives in our cohort suggests that household and peri-domestic outdoor activities, such as gardening and routine chores around the home, may represent an important exposure route for women. Similar patterns have been reported in northern Iran and Brazil, where domestic or peri-domestic environments were identified as relevant settings for Hymenoptera exposure [3,8,11,15,17]. Furthermore, studies from Thailand and Sri Lanka reinforce the observation that individuals involved in agriculture and manual labor face the highest risk due to increased occupational exposure in outdoor environments where stinging insects are common [7,20]. These results indicate that preventive strategies in the region should be dual-focused: emphasizing the use of protective equipment for outdoor manual workers (predominantly males) and raising awareness about habitat management in domestic environments for housewives.

Urban-rural exposure patterns are strongly influenced by species ecology and local settlement structure. Africanized honeybees, for example, have increasingly colonized urban environments, as documented in Brazil, where Linard et al. [12] found that nearly 78% of stings occurred in cities due to nesting in walls and ornamental trees. In contrast, our study showed only a slight urban predominance (53.3%), suggesting a mixed exposure pattern rather than a clearly urban-centered distribution. Conversely, Xie et al. in China [13] reported that 85% of victims were rural residents, where dense wasp populations and occupational contact contributed to multiple severe stings. Taken together, these findings indicate that the urban-rural burden of Hymenoptera stings depends largely on species ecology, land use, and settlement characteristics.

Our study documented a summer peak (52.4%) with a secondary rise in autumn (26.7%). Bemanian et al. in Gorgan, Iran, also reported a summer predominance, citing Hymenoptera activity during flowering and increased human outdoor exposure [11]. By contrast, Himral et al. in Northern India found a peak in autumn (October–December), which they linked to intensified wasp foraging when food becomes scarce [9]. These observations demonstrate how climate, flowering cycles, and species-specific behavior shape seasonal sting incidence.

In our study, head and neck stings were most common (44.9%). Reports from other regions illustrate varied anatomical distributions. In Switzerland, Braun et al. [14] observed high rates of craniofacial and intraoral stings, considered particularly dangerous due to airway compromise. In Taiwan, Yu et al. [19] noted a predominance of limb involvement, likely due to exposed extremities during daily activities. Together, these data suggest that cultural habits, such as clothing coverage, and the defensive targeting of elevated body parts by Hymenoptera significantly influence sting localization [7,8,16,17,21]. Importantly, our findings revealed a highly significant gender-based disparity in the anatomical distribution of stings. While men predominantly suffered stings on the head and neck (51.6% vs. 28.1% in women), women experienced a significantly higher proportion of stings on their hands (35.9%) and legs (29.7% vs. 4.3% in men). This divergence may be partly influenced by common regional clothing patterns and gender-specific activities. In many public and outdoor settings in Iran, women commonly wear headscarves and long garments that cover the hair, neck, and torso. Such clothing may function as a physical barrier that reduces exposure of the head, neck, and upper body to insect contact. Conversely, areas that remain more exposed during household or agricultural tasks, such as the hands and lower limbs, may become more vulnerable to stings. For men, outdoor work with relatively greater exposure of the head, neck, and upper extremities may contribute to the higher frequency of craniofacial stings. This interpretation should be considered hypothesis-generating and warrants confirmation in future studies with more detailed exposure data [13]. This demonstrates that socio-cultural clothing norms, alongside gender-specific occupational and domestic roles, play a pivotal role in dictating the anatomical micro-environments of Hymenoptera envenomation.

Mechanistically, Hymenoptera venom elicits IgE-mediated hypersensitivity reactions, explaining the spectrum of local and systemic symptoms described worldwide [6]. Our study identified both local and systemic reactions, paralleling Bemanian et al. [11], who reported skin and respiratory involvement as dominant. However, Witharana et al. in Sri Lanka [20] found that even patients with over 100 stings usually developed only mild or moderate reactions, attributing this to species differences in venom potency. Such contrasts reinforce that host response, venom composition, and species ecology collectively determine clinical severity.

In our study, 28.0% of patients required hospitalization. The burden was far higher in China, where Xie et al. [13] reported frequent multi-organ dysfunction and a 5.1% mortality rate, attributed to multiple stings and delayed presentation averaging 10 hours. At the other extreme, Linard et al. in Brazil [12] noted that more than 80% of cases were mild, with deaths occurring only after treatment delays. These comparisons underscore that clinical severity reflects not only venom toxicity but also the number of stings and the timeliness of care.

Global evidence points to a persistent gap in evidence-based management. In Switzerland, Braun et al. [14] reported widespread use of antihistamines (97%) and corticosteroids (80%), but epinephrine was given in only 12% of patients, even in severe cases. In our study, corticosteroids were the most frequently administered treatment (75.0%). This is highly consistent with global clinical practices reported in a systematic review by Liyanage et al., which found that the average rate of corticosteroid use in the emergency management of anaphylaxis and severe allergic reactions is approximately 68%, ranging up to 100% in some settings [22]. The high utilization rate in our cohort may reflect local clinical practice patterns aimed at managing inflammatory responses and potential airway edema, especially given the high frequency of head and neck stings observed in our patients. Improved clinical documentation, provider training, and standardized treatment protocols are essential to strengthen evidence-based management. In Iran, Bemanian et al. [3,11] described a similar, concerning underutilization of epinephrine, which they attributed to inadequate physician awareness and fear of adverse effects. By contrast, guideline adherence was stronger in Thailand, where Charoenwikkai et al. [7] documented epinephrine use in 77% of anaphylaxis cases. Together, these findings suggest that corticosteroids are frequently used in the acute management of Hymenoptera sting reactions, while previous studies have reported variable adherence to first-line epinephrine use in anaphylaxis. Standardized protocols and provider education may help improve evidence-based emergency care. The lower utilization of antihistamines (31.6%) compared to corticosteroids (75.0%) in our study suggests that clinical management was primarily focused on preventing severe inflammatory complications and airway compromise rather than the symptomatic relief of cutaneous reactions.

Although our retrospective data categorized stings into broad anatomical regions (e.g., head and neck) and did not specifically delineate intraoral stings within these records, it is clinically pertinent to emphasize the risk of such injuries. As highlighted in studies by Braun et al., intraoral stings [14], which often occur due to accidental ingestion of insects, represent a unique emergency scenario. Even if not explicitly captured in our current registry, clinicians should remain highly vigilant of this possibility when treating patients with head and neck stings. The anatomical vulnerability of the oropharynx means that even minor inflammatory responses in this region can progress rapidly to airway obstruction, necessitating close monitoring and early, aggressive intervention.

Taken together, the global evidence indicates that Hymenoptera sting epidemiology is shaped by ecological conditions, occupational exposure, and cultural practices, with consistent male predominance in most regions but important exceptions in specific contexts [9,19]. A strength of the present investigation is the inclusion of a broad clinical spectrum of sting victims across multiple years and settings, which enabled reliable assessment of seasonal and demographic patterns. Nonetheless, inherent limitations of retrospective design, the absence of species-level identification, and lack of follow-up data restrict the generalizability of the findings, as noted in other regional reports [2,12]. Clinically, previous studies have reported variable and sometimes suboptimal use of epinephrine in anaphylaxis management, highlighting the need for improved documentation, standardized emergency protocols, and provider training [7,11,14].

## Limitations

Species-level identification of Hymenoptera was not consistently available in medical records because insects were generally identified based on patients’ descriptions rather than entomological confirmation, limiting species-specific analysis. Regarding the generalizability of our findings, this study focused on three major counties in East Azerbaijan province. While these areas provide a representative snapshot of the pediatric and adult populations in northwestern Iran due to their diverse urban and rural demographics, the results may not be directly generalizable to regions with significantly different climates, Hymenoptera species distributions (e.g., southern coastal areas), or different socio-cultural practices. Furthermore, the small number of patients requiring prolonged hospitalization (>24 hours, n = 4) limits the statistical power of subgroup analyses comparing clinical symptoms between short-term and long-term hospital stays.

## Conclusion

Hymenoptera stings remain a significant public health concern with considerable variability in demographic, seasonal, and clinical patterns across regions. Our findings highlight a predominance among males, a bimodal age distribution, summer peaks, and frequent head and neck involvement, reflecting the interplay of ecological, occupational, and cultural factors. Preventive strategies should address both domestic and occupational exposure settings, including habitat management in residential areas, public awareness, and protective clothing for outdoor workers. Strengthening provider training and implementing standardized emergency treatment protocols may help improve evidence-based care, including the appropriate use of epinephrine in suspected anaphylaxis. Future research should adopt prospective multicenter designs, integrate entomological surveys, and evaluate preventive interventions tailored to high-risk populations.

## References

[1] Da Silva LOP, Mendonça PM, Cortinhas LB, Ribeiro PC, Norberg AN, Norberg PRB. Increasing incidence of bee stinging in Brazil: an epidemiological study. J Adv Med. 2024;36(8):188–203.

[2] Feás X, Vidal C, Remesar S. What We Know about Sting-Related Deaths? Human Fatalities Caused by Hornet, Wasp and Bee Stings in Europe (1994-2016). Biology (Basel). 2022;11(2):282. doi: 10.3390/biology11020282 35205148 PMC8869362

[3] Bemanian MH, Ghelichi-Ghojogh M, Aghapour SA. A New Approach to Eliminate Hymenoptera Venom Grading Sensitization Test in the North Iran: Cross-Sectional Study. Med J Islam Repub Iran. 2024;38:2. doi: 10.47176/mjiri.38.2 38434225 PMC10907046

[4] Sedighi G, Dehghani R, Varzandeh M. Toxic reaction of a 3-year-old boy due to Hornet multiple stings in Kerman-Iran province: A case report. Toxicon. 2023;221:106976. doi: 10.1016/j.toxicon.2022.106976 36403779

[5] Dehghani R, Fathi B, Dehghani M, Mohammadzadeh N. Venomous and poisonous arthropods in Iran, West Asia, and the Middle East: an overview of their identification, bites, stings, behavior, biology and geographical distribution. Iran J Vet Sci Technol. 2025;17(1):1–36.

[6] Serrano Sanchez T, Guirola Fuentes J, Mastrapa Ochoa H, Batista Reyes Y, Jomarron Martinez Y, Pelaez Rodriguez J. Bee and wasp stings cause type I hypersensitivity reactions, mechanism and treatment. Asia Pac J Med Toxicol. 2023;11(4):163–5.

[7] Charoenwikkai S, Intapun P, Lao-Araya M. Bee sting injuries in Thailand’s high apicultural area: Outcome, risk and treatment patterns. Risk Manag Healthc Policy. 2024;17:1837–45. 39050091 10.2147/RMHP.S470007 PMC11268765

[8] Diniz AGQ, Belmino JFB, de Araújo KAM, Vieira AT, Leite RS. Epidemiology of honeybee sting cases in the state of ceará, northeastern Brazil. Rev Inst Med Trop Sao Paulo. 2016;58:40. doi: 10.1590/S1678-9946201658040 27253742 PMC4879997

[9] Himral PH. Clinical profile of Hymenoptera sting in a tertiary care hospital in Himachal Pradesh. J Evol Med Dent Sci. 2018;7(43):4675–7.

[10] Senthilkumaran S, Balamurugan N, Karthikeyan N, Thirumalaikolundusubramanian P. Honey Bee Stings: Historical, Clinical, Toxicological, and Environmental Aspects. Am J Med. 2020;133(6):e321. 10.1016/j.amjmed.2020.01.013 32532377

[11] Bemanian MH, Arshi S, Nabavi M, Fallahpour M, Aarabi M, Karbasi M. Anaphylactic reaction to bee stings in the rural areas of Gorgan City: Iran’s first epidemiological study of Hymenoptera-induced anaphylaxis. J Pediatr Rev. 2019;7(4):239–48.

[12] Linard AT, Barros RM, Sousa JA, Leite RS. Epidemiology of bee stings in Campina Grande, Paraíba state, Northeastern Brazil. J Venom Anim Toxins Incl Trop Dis. 2014;20:13. 24694193 10.1186/1678-9199-20-13 PMC3997214

[13] Xie C, Xu S, Ding F, Xie M, Lv J, Yao J, et al. Clinical features of severe wasp sting patients with dominantly toxic reaction: analysis of 1091 cases. PLoS One. 2013;8(12):e83164. doi: 10.1371/journal.pone.0083164 24391743 PMC3877022

[14] Braun CT, Mikula M, Ricklin ME, Exadaktylos AK, Helbling A. Climate data, localisation of the sting, grade of anaphylaxis and therapy of Hymenoptera stings. Swiss Med Wkly. 2016;146:w14272. doi: 10.4414/smw.2016.14272 26859128

[15] de Araújo KAM, de Araújo JMD, de Souza Leite R. Epidemiological study of the bee stings in the state of Bahia, northeastern Brazil, from 2010 to 2019. Rev Ciênc Méd Biol. 2022;21(1):73–8.

[16] Marques MRV, Araújo KAM, Tavares AV, Vieira AA, Leite RS. Epidemiology of envenomation by Africanized honeybees in the state of Rio Grande do Norte, Northeastern Brazil. Rev Bras Epidemiol. 2020;23:e200005. 10.1590/1980-549720200005 32130394

[17] Araújo JMD, Sousa JA, Vieira AA, Leite RS. Epidemiological profile of the bee stings in piauí, northeastern Brazil, from 2011 to 2020. Educ Ci e Saúde. 2025;12(1). doi: 10.20438/ecs.v12i1.630

[18] Trangadia M, Kharadi R, Gupta B. Epidemiologic study of fatal and non-fatal poisoning case in pediatric, around Jamnagar region, Gujarat in India (January-December 2013). Int J Med Toxicol Forensic Med. 2016;6:3.

[19] Yu C-H, Tan S-T, Yang H-W, Lai Y-C, Su Y-J. Gender-Based Clinical Differences in Hymenoptera Venom Poisoning: A Retrospective Study From Taiwan (April 2021 to March 2023). Emerg Med Int. 2025;2025:8893175. doi: 10.1155/emmi/8893175 40495953 PMC12149513

[20] Witharana EWRA, Wijesinghe SKJ, Pradeepa KSM, Karunaratne WAIP, Jayasinghe S. Bee and wasp stings in Deniyaya; a series of 322 cases. Ceylon Med J. 2015;60(1):5–9. doi: 10.4038/cmj.v60i1.7406 25804910

[21] Lee JH, Kim MJ, Park YS, Kim E, Chung HS, Chung SP. Severe Systemic Reactions Following Bee Sting Injuries in Korea. Yonsei Med J. 2023;64(6):404–12. doi: 10.3349/ymj.2022.0532 37226567 PMC10232995

[22] Liyanage CK, Galappatthy P, Seneviratne SL. Corticosteroids in management of anaphylaxis; a systematic review of evidence. Eur Ann Allergy Clin Immunol. 2017;49(5):196–207. doi: 10.23822/EurAnnACI.1764-1489.15 28884986

